# The Neuro-Mechanical Processes That Underlie Goal-Directed Medio-Lateral APA during Gait Initiation

**DOI:** 10.3389/fnhum.2016.00445

**Published:** 2016-08-31

**Authors:** Jean-Louis Honeine, Marco Schieppati, Oscar Crisafulli, Manh-Cuong Do

**Affiliations:** ^1^Department of Public Health, Experimental and Forensic Medicine, University of PaviaPavia, Italy; ^2^Centro Studi Attività Motorie (CSAM), Fondazione Salvatore Maugeri (IRCSS), Scientific Institute of PaviaPavia, Italy; ^3^Faculty of Sport Science, Complexité, Innovations, Activités Motrices et Sportives (CIAMS), Université Paris-SaclayOrsay, France

**Keywords:** gait initiation, center of pressure, center of mass, frontal plane, knee flexion, ankle dorsiflexor muscles, hip abductor muscles

## Abstract

Gait initiation (GI) involves passing from bipedal to unipedal stance. It requires a rapid movement of the center of foot pressure (CoP) towards the future swing foot and of the center of mass (CoM) in the direction of the stance foot prior to the incoming step. This anticipatory postural adjustment (APA) allows disengaging the swing leg from the ground and establishing favorable conditions for stepping. This study aimed to describe the neuro-mechanical process that underlies the goal-directed medio-lateral (ML) APA. We hypothesized that controlled knee flexion of the stance leg contributes to the initial ML displacement of the CoP and to the calibration of the first step. Fourteen subjects initiated gait starting from three different initial stance widths of 15 cm (Small), 30 cm (Medium), and 45 cm (Large). Optoelectronic, force platform and electromyogram (EMG) measurements were performed. During APA, soleus activity diminished bilaterally, while tibialis anterior (TA) activity increased, more so in the stance leg than in the swing leg, and to a larger extent with increasing initial stance width. Knee flexion of the stance leg was observed during APA and correlated with the ML CoP displacement towards the swing leg. ML CoP and CoM displacements during APA increased with increasing stance width. The activity of stance-leg TA was correlated with the degree of knee flexion. Swing-leg tensor fasciae latae (TFL) was also active during APA. Across subjects, when stance-leg tibialis activity was low, TFL activity was large and vice versa. The modulation of the ML CoP position during APA allowed the gravity-driven torque to place the CoM just lateral to the stance foot during step execution. Accordingly, the gravity-driven torque, the ML CoM velocity during step execution, and the step width at foot contact (FC) were lower in the Small and greater in the Large condition. Consequently, the position of the stepping foot at FC remained close to the sagittal plane in all three conditions. Conclusively, coordinated activation of hip abductors and ankle dorsiflexors during APA displaces the CoP towards the swing leg, and sets the contact position for the swing foot.

## Introduction

Gait initiation (GI) is a critical task because it involves transiting from stable bipedal stance to unstable unipedal stance. It requires anticipatory postural adjustments (APA) that reconfigure the position of the center of mass (CoM) with respect to the center of foot pressure (CoP) in order to take advantage of gravity and progress forward (Carlsöö, 1966; Brenière and Do, 1986, 1991; Brenière et al., 1987; Brenière, 2001).

It is well established that the displacement of CoM during APA is achieved by shifting the CoP backwards and in the direction of the swing leg (Jian et al., 1993; Elble et al., 1994; Lepers and Brenière, 1995). The backward shift of the CoP is caused by a reduction in soleus activity and activation of the tibialis anterior (TA; Crenna and Frigo, 1991). During APA, modulation of the antero-posterior distance between the vertical projections to the ground of the CoM and CoP positions generates a torque driven by gravity (henceforth termed disequilibrium torque) that propels the body forward (Lepers and Brenière, 1995; Michel and Do, 2002; Honeine et al., 2013, 2014). In the frontal plane, the initial CoP excursion in the direction of the future swing leg during APA causes gravity to move the CoM closer to the stance leg (Jian et al., 1993; Elble et al., 1994; Lepers and Brenière, 1995; McIlroy and Maki, 1999). By doing so, the nervous system disengages the swing leg from the ground. During the single-support phase of the first step, the distance between CoM and CoP in the frontal plane and the mediolateral (ML) fall of the CoM shape the positioning of the stepping foot at the moment of its contact with the ground (Lyon and Day, 1997, 2005; Caderby et al., 2014).

Carlsöö (1966) and later Winter (1995) stated that the initial displacement of the CoP in the direction of the swing leg is caused by unloading the stance leg and loading the swing leg, which they argued is produced by the hip abductor activity from the swing side. However, this may not be the sole mechanisms underpinning the disengagement of the future swing limb. When standing, the knee joint is extended due to the backward pull of the soleus on the tibia and to the action of gravity (Woodhull et al., 1985) connected with the anterior position of the CoM with respect to the knee. Therefore, the knee acts as a load-bearing joint (Walker and Erkman, 1975; Freeman et al., 1980). Notably, flexion of the knee of the stance leg during single-support is known to occur during gait (Segal et al., 2006; Shamaei et al., 2013). Hence, it may be appropriate to examine whether flexion of the knee also occurs during the APA in order to unload the stance leg during the preparation phase of GI. In this study, we tested the hypothesis that the central nervous system (CNS) employs stance-leg knee flexion as a complementary strategy to swing-leg hip abduction in order to unload the stance leg and displace the CoP in the direction of the swing leg during APA. Furthermore, we postulated that the modulation of the CoP displacement during APA also determines the disequilibrium torque and the lateral fall of the body towards the swing leg during step execution, with the purpose of positioning the stepping foot close to the sagittal plane at its foot contact (FC).

We asked volunteers to start walking from different initial stance widths at their spontaneous forward velocities. Increasing the stance widths prior to step execution has been shown to produce larger CoM and CoP displacements, in order to modulate the distance between CoM and CoP in the frontal plane and consequently the gravity-driven torque during step execution (Lyon and Day, 1997). If our hypothesis is correct, flexion of the knee of the stance leg should occur in the APA phase. The finding of a greater knee flexion of the stance leg accompanied by larger CoM and CoP displacement with a larger initial stance width would corroborate the proposition.

We also expected the ampler CoP displacement, which should occur the larger the initial stance width, to produce a greater gravity-driven torque during step execution. This would in turn increase the velocity of the CoM fall in the frontal plane prompting the stepping foot to land close to the subjects’ sagittal plane.

## Materials and Methods

### Participants

Fourteen (11 F) healthy young adults participated in the study. Their mean age, mass and height were 29 years (ranging from 21 to 41), 61 kg (47–80) and 1.68 m (1.58–1.80), respectively. As conformed to the Declaration of Helsinki, subjects provided written informed consent to the experiment, which was approved by the ethics committee of Fondazione Salvatore Maugeri (No. 2056CE).

### Tasks and Procedures

Subjects stood with each foot on a distinct force platform (Kistler 9286BA, Winterthur, Switzerland). The position of each heel was equidistant to the border of each platform. Following the start of the acquisition, subjects were instructed to initiate walking in a self-paced mode. This was done to avoid the startle effect on GI, which occurs when subjects are asked to start walking following an acoustic command (Queralt et al., 2010). Subjects were not given any instruction about the speed at which to initiate walking and were left to execute GI at their preferred velocity. They performed three sets of conditions of GI, in which they started from predefined initial stance-widths of 15, 30 and 45 cm, termed Small, Medium and Large, respectively, in the text. There was no explicit instruction about the positioning of the first or successive footsteps. The order of the trials was randomized across subjects. Following GI, subjects were instructed to continue walking for at least six steps. Subjects were not specifically told to execute GI starting from a given leg (Hiraoka et al., 2014), even if in most of the trials subjects initiated gait with the same leg. Subjects performed as many trials as needed in order to achieve at least 12–15 trials per condition, all performed with the same leg. The distribution of the weight over each leg was checked during the experiment so that if the difference exceeded 1 kg before the CoP started moving, the trial was repeated in order to prevent the effects of anticipation on the GI features.

### Data Acquisition

Twenty-three reflective markers were positioned on the subjects’ body in the following anatomical locations: vertex, acromion, C7, L5 and bilaterally on lateral head, medial epicondyle of the elbow, head of the ulna, anterior superior iliac spine, greater trochanter, lateral epicondyle of the femur (knee), lateral malleolus, heel and 1st metatarsus-phalangeal joint. The Smart-D optoelectronic system (BTS Bioengineering, Italy) employed 12 infrared cameras that measured the position of the body markers with respect to the laboratory global reference. Anthropometric measurements of the segments of the body were made manually. This allowed for the computation of the three-dimensional position of the body’s CoM (Winter, 1993). Bipolar electrodes (Freeemg, BTS Bioengineering, Italy) were employed to wirelessly record bilaterally the electromyogram (EMG) of TA, soleus, gastrocnemius medialis, biceps femoris (BF), semitendinosus (ST), semimembranosus (SM), tensor fasciae latae (TFL) and gluteus medius (nine subjects). Skin preparation and electrode placement were performed according to the SENIAM protocol (Merletti and Hermens, 2000). Optoelectronic, force platform and EMG data were recorded synchronously at acquisition frequencies of 140, 560, 1000 Hz, respectively. Data were stored in a computer for off-line analysis using a custom-made program written using Matlab (Mathworks, Natick, MA, USA).

### Analysis of Biomechanical Variables

The global ML CoP position was calculated from the output of both force platforms according to Equation 1:

(1) $${\text{CoP}}_{\text{global}}\;\text{=}\;\left[\left({\text{F}}_{1}\;\text{*}\;{\text{CoP}}_{\text{1}}\right)\;\text{+}\;\left({\text{F}}_{2}\;\text{*}\;{\text{CoP}}_{\text{2}}\right)\right]\text{/}\left({\text{F}}_{1}\;\;\text{+}\;{\text{F}}_{2}\right)$$

where F_1_ and F_2_ are the vertical ground reaction forces under each leg, and CoP_1_ is the ML position of CoP under one leg while CoP_2_ is the ML position of the CoP under the other. The coordinates of CoP_1_, CoP_2_, CoP_global_ and the positions of each marker were referred to the same laboratory reference system.

**Figure 1:**
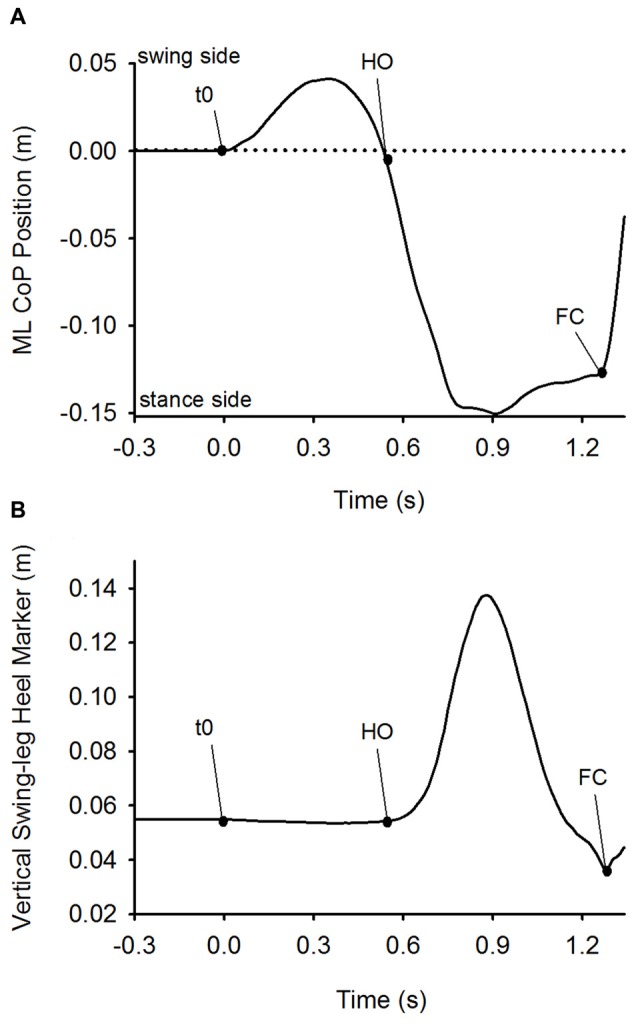
**Detection of t0, heel-off (HO) and foot-contact (FC).** Panel **(A)** depicts the time-course of the mediolateral (ML) center of foot pressure (CoP) position. Panel **(B)** shows the time-course of the vertical position of the heel marker of the swing leg. Both plots were obtained from a single trial of one subject in the Medium stance-width condition. The dots on the curves indicate the time of initiation of CoP movement (t0), HO and FC. Anticipatory postural adjustment (APA) was defined as the time-period between t0 and HO, and step execution as the time-period between HO and FC.

The instant at which GI effectively started (t0) was set as the instant at which the ML CoP position exceeded the baseline by three standard deviations. The instant of the first heel-off (HO) was determined when the vertical position of the heel marker of the swing leg exceeded the value at baseline by 1 mm. The instant when the heel marker of the swing-leg reached its lowest vertical position signaled the instant of the successive FC (see Figure 1). Both instants were referred to t0. APA was defined as the time-period spanning from t0 to HO. The step execution phase extended from HO until swing foot landing (FC). The velocity of the CoM in the frontal plane was obtained by the derivation of the ML component of CoM position in time. Step length at FC was measured as the distance between the position of the heel of the stepping foot at t0 and at FC in the sagittal plane. Step width at FC was the distance in the frontal plane between the stance-foot heel marker at t0 and stepping-foot heel marker at its FC. The greater trochanter, knee and malleolus markers on both sides were used to calculate the knee angles in the sagittal plane.

The unloading of the stance leg was measured as the reduction of vertical ground reaction force at the instant of maximum CoP displacement during APA with respect to that measured at t0. The loading of the swing leg during APA was measured as the amount of increase in vertical ground reaction force at the instant of maximum CoP displacement during APA with respect to that measured prior to t0.

The instantaneous disequilibrium torque acting in the frontal plane was calculated as the product of the vertical ground reaction force and the distance between the ground vertical projection of CoM and CoP in the frontal plane (Equation 2). To calculate the torque, the CoP position and the vertical force were down-sampled to 140 Hz. Due to friction between the foot and the floor, the disequilibrium torque is converted to shear force. In order to calculate the shear component of the ground reaction force generated by the torque, the latter was divided by the instantaneous height of CoM with respect to CoP (Equation 3):

(2) Torque  =  d(CoM-CoP)* F

(3) Shear force = Torque/h

where d(CoM−CoP) is the distance between CoM and CoP in the frontal plane, F is the vertical force acting on the CoM and h is the vertical position of CoM with respect to the ground. The impulse during APA and during step execution was measured as the product of the average shear force during APA or during step execution and the respective time periods in which the force was applied (Equation 4). The impulse was then normalized by dividing it by the corresponding subject’s mass.

(4) $$\text{Impulse}\;\text{=}\;\text{Shear}\;{\text{force}}_{\text{Avg}}\;\text{*}\;\Delta \text{t}$$

### Analysis of EMG Activity

**Figure 2:**
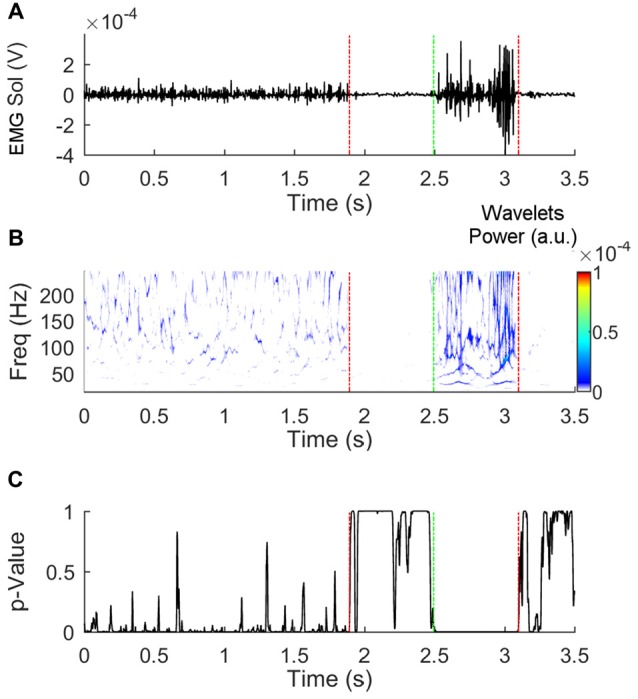
**Detection of the beginning and end of electromyogram (EMG) activity.** Panel **(A)** shows a raw EMG trace obtained from the soleus muscle of the stance leg during Gait initiation (GI). Panel **(B)** represents the synchrosqueezed transform of the raw trace. The *p*-values of the *t*-test that compares the coefficients in each time-bin with the control-matrix-coefficients are shown in panel **(C)**. Fifty or more consecutive *p*-values inferior to 0.05 indicates that the muscle is active. The vertical dashed lines indicate the instants at which deactivation (red) and activation (green) were detected.

To calculate the start and end of EMG activities, the synchrosqueezed wavelet transforms of the raw EMG traces were calculated using the Morlet wavelet (Daubechies et al., 2011; Iatsenko et al., 2015). The final wavelet transform was composed of 125 frequency bins with a bandwidth of about 1.64 Hz ranging from 40 to 244.8 Hz for each time frame. A control-wavelet-coefficient matrix (154 × 1) was then created by averaging the coefficients that were found in the 25th percentile of the coefficient distribution. Multiple single-tailed Student’s *t*-test was then used to compare each time-frame of the wavelet transform with the control-wavelet-coefficient matrix. Onset of muscle activation was considered the instant when *p*-value exceeded 0.05 for 50 consecutive time-frames. Offset was considered the instant when *p*-value was inferior to 0.05 for 50 consecutive time-frames (see Figure 2). The amplitude of EMG activity was calculated by rectifying the EMG raw traces, low-pass filtering the signal with a second order Butterworth no-lag filter with a cut-off frequency of 25 Hz, and calculating the area under the curve. Maximal EMG isometric activity of all muscles was measured by blocking the pertinent body segment against a rigid frame and asking the subject to exert a maximal isometric contraction against it for about 3–4 s. The peak activity of EMG was detected. The amplitude of the EMG activity during a time-window of 1 s centered around the peak activity was then calculated.

### Statistics

We verified the distribution of each parameter by performing a Shapiro-Wilk test. Parameters that did not show a normal distribution were tested using non-parametric methods. Separate repeated-measures ANOVAs were used to test the effect of initial stance-width on the following variables: duration of APA; duration of step execution; anteroposterior (AP) instantaneous velocity of CoM at HO and at FC; maximum distance between CoP and CoM during APA and during step execution; EMG activities of stance-leg soleus, gastrocnemius, TFL and gluteus medius during step execution; position of the swing-leg heel marker at FC; position of the swing-leg hallux marker at FC. Separate 2 (swing − stance) × 3 (Small − Normal − Large) repeated-measures ANOVAs were used to test the statistical difference of the following variables: forward displacement of knee joints during APA; knees flexion during APA. Separate one-way Friedman repeated-measures analyses were used to test, during APA, differences in the amplitude of the swing- and stance leg TA EMG activity; effect of initial stance-width on the activity of the swing-leg TA activity; effect of initial stance-width on the activity of stance-leg tibialis activity; effect of initial stance-width on the activity of the swing-leg TFL. Pearson’s method was used to test the linear relationship between the following variables: unloading and loading of the stance and swing legs, respectively, and the maximum displacement of CoP during APA; stance-leg knee forward displacement and stance-leg knee flexion; stance-leg knee flexion and maximum displacement of CoP during APA; amplitude of TA activity and knee flexion of the stance leg during APA; normalized impulse calculated during APA and normalized impulse during step execution; normalized impulse during step execution and ML CoM velocity at FC; normalized impulse during step execution and step width at FC. Hyperbolic fitting was used to determine the relationship between the TFL and TA activity in each of the three conditions. Linear and hyperbolic relationship were done using Sigmaplot (Systat software Inc., San Jose, CA, USA). *Post hoc* analyses of variables following a normal distribution were made by the Fischer’s LSD test. The Wilcoxon signed-rank test was used to test non-normally distributed variables. The level of significance in all tests was set at *p* < 0.05. The software Statistica (StatSoft, Tulsa, OK, USA) was used.

## Results

### Global Kinematics

**Figure 3:**
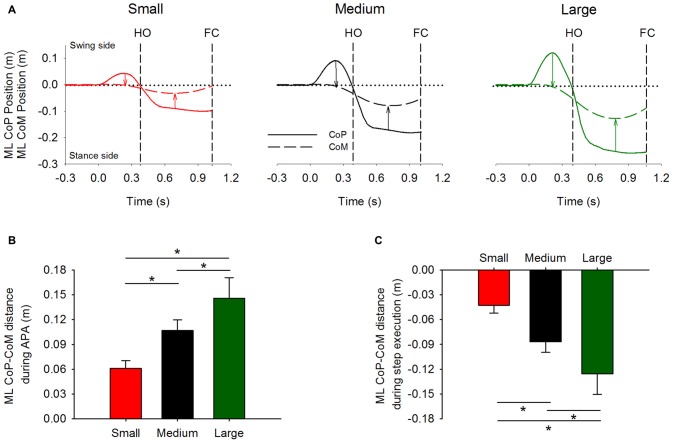
**Global kinematics variables in the frontal plane.** The average time-course obtained from a single subject of the ML CoP (solid line) and center of mass (CoM) position (dashed line) are shown in panel **(A)**. The vertical dashed lines indicate HO and FC events. Panels **(B,C)** show the grand means and standard deviations of the distance between CoP and CoM at the instant at which CoP displacement peaked during APA (indicated by the leftmost arrows in panel **A**), and of the distance between CoP and CoM at the instant at which CoM displacement peaked during step execution (indicated by the rightmost arrows in panel **A**), respectively. *Indicates significant difference (*p* < 0.05).

As expected, prior to stepping, CoP was initially displaced in the direction of the swing leg while CoM moved in the opposite direction. Figure 3A shows the time-course of the mean curves of the ML CoP and CoM positions in the three stance-width conditions of a single subject (mean curves were obtained by averaging 15 trials). The instant *t* = 0 s, which is referred to as t0 in the text, indicates the instant at which the ML CoP position starts moving towards the swing leg. As can be seen in the Figure, prior to HO, the CoP is displaced towards the swing leg and the CoM is moved, with a delay, in the direction of the stance leg. Then, the CoP starts moving towards the stance leg. Next, around mid-stance, the CoM lateral excursion peaks and starts moving towards the future stance leg in anticipation of the second step.

The distance between CoP and CoM in the frontal plane was measured at two distinct moments: the instant when CoP lateral excursion peaks during APA and the instant when CoM reaches its maximum displacement during step execution (as indicated by the arrows in Figure 3A). Their grand means and standard deviations are shown in Figures 3B,C. The ANOVA showed a significant effect of the initial stance-width on the maximal distance between CoP and CoM during APA (*F*_(2,26)_ = 156.6, *p* < 0.001), and on the maximal distance between CoP and CoM during step execution (*F*_(2,26)_ = 153, *p* < 0.001). *Post hoc* analyses showed that the maximal distance between CoP and CoM during APA and during step execution were the lowest in the Small and the greatest in the Large stance-width condition (*p* < 0.001 for all comparisons). The CoP displacement during APA was not significantly correlated with the subjects’ height (Small: *r*^2^ = 0.09, *p* = 0.31; Medium: *r*^2^ = 0.04, *p* = 0.48; Large: *r*^2^ = 0.06, *p* = 0.41), or their pelvis width (Small: *r*^2^ = 0.13, *p* = 0.21; Medium: *r*^2^ = 0.07, *p* = 0.36; Large: *r*^2^ = 0.01, *p* = 0.7).

Table 1 contains the grand mean and standard deviation of the duration of APA, the duration of step execution, the instantaneous forward velocity of the CoM at the time instant of HO, and the forward velocity of CoM at the time instant of FC. Repeated-measures ANOVA showed no effect of initial stance width on the duration of APA (*F*_(2,26)_ = 2.5, *p* = 0.1). The instantaneous forward velocity of CoM measured at the instant of HO was also comparable between the three conditions (*F*_(2,26)_ = 1.1, *p* = 0.35). The ANOVA showed an effect of initial stance width on the duration of step execution (*F*_(2,26)_ = 11, *p* < 0.001) and the instantaneous forward CoM velocity measured at the instant of FC (*F*_(2,26)_ = 14.7, *p* < 0.001). *Post hoc* analysis showed that both duration of step execution and forward velocity of CoM at FC were smaller in the Large stance-width condition with respect to the Small and Medium conditions (*p* < 0.001).

**Table 1:** **Global gait initiation (GI) variables**.

Condition	Duration of APA (s)	Duration of step execution (s)	CoM velocity at Heel-off (ms^-1^)	CoM velocity at foot-contact (ms^-1^)
Small	0.763 ± 0.06	0.635 ± 0.07	0.12 ± 0.03	0.81 ± 0.14
Medium	0.765 ± 0.05	0.629 ± 0.06	0.12 ± 0.04	0.80 ± 0.14
Large	0.784 ± 0.06	0.581 ± 0.07*	0.11 ± 0.05	0.74 ± 0.16*

*Indicates significant difference, p < 0.05.

### CoP ML Displacement and Flexion of the Knee of the Stance Leg

**Figure 4:**
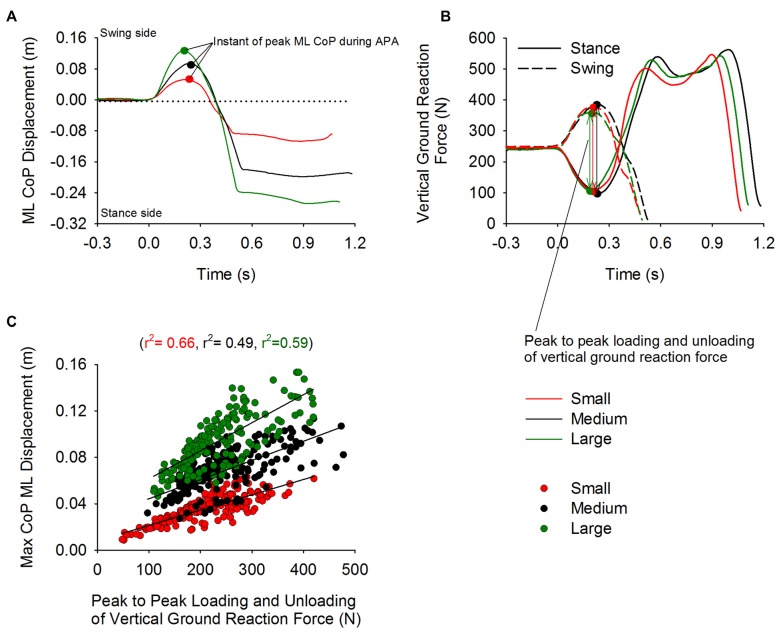
**Unloading and loading of the stance and swing leg and CoP displacement during APA.** Panels **(A,B)** show the time-course of the CoP displacement and the vertical reaction force under the swing (dashed line) and stance foot (solid line), respectively. The curves were obtained from trials of a single subject from the Small, Medium and Large condition. The small circles on the plot indicate the instant at which the displacement of CoP in the direction of the swing leg peaked during APA. Panel **(C)** shows the three distinct linear relationships (one for each condition) that were found between maximal CoP displacement and the unloading and loading (measured peak to peak) of the stance and swing leg, respectively.

Figure 4 shows the time-course of the ML CoP position (panel **A**) and the vertical ground reaction force (Panel **B**) under each foot. The traces represent a single trial obtained in each of the three stance-width conditions (Small, Medium and Large). The dots on the curves represent the moment at which the CoP displacement peaked. The loading of the swing and the unloading of the stance leg were accompanied by the displacement of CoP in the direction of the swing leg. Figure 4C shows a scatter plot of the maximum displacement of CoP as a function of the sum of the unloading and loading of the stance and swing leg (measured peak to peak). Three distinct linear relationships were obtained between the unloading of the stance leg and the maximum displacement of CoP during APA (Small: *r*^2^ = 0.66, Medium: *r*^2^ = 0.49, and Large: *r*^2^ = 0.59). The relationships diverge because the position of the CoP in the frontal plane is not only caused by the amplitude of the vertical ground reaction force under each leg, but is also dependent on the initial stance width (see Equation 1).

**Figure 5:**
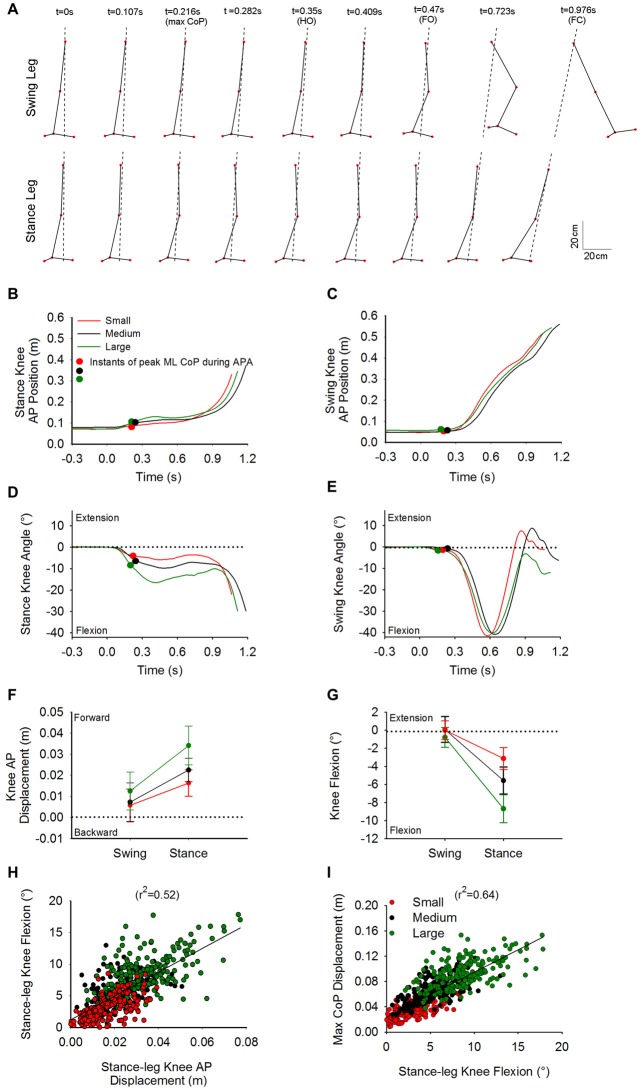
**Knee flexion and CoP displacement during APA.** Panel **(A)** shows stick diagrams of the swing leg (top) and stance leg (bottom) taken at nine discrete instants in the sagittal plane. The stick diagrams represent the same Medium-condition trial as in Figure 4. The dashed lines represent the direction of the CoP-CoM vector. The instants comprise t0, instant of peak ML CoP during APA, HO, foot-off, FC and instants in between. As can be seen at t0, both knees are positioned behind the center of gravity. During APA, the swing-leg knee remains fixed while the ankle joint starts flexing and the stance-leg knee advances ahead of the center of gravity. The time-course of the stance knee position and swing knee position for the three single trials in each condition are found in panels **(B,C)** respectively. Panels **(D,E)** show the time-course of the stance-leg and swing-leg knee angle, respectively. The curves in panels **(B–E)** belong to the same trial. The small circles on the curves indicate the instant at which the maximum displacement of CoP in the direction of the swing leg was reached during APA. Panels **(F,G)** show the grand means and standard deviations of the forward displacement and flexion of the knee of the swing and stance leg, respectively, measured at the instant at which CoP displacement peaked. Panels **(H,I)** portray the linear relationship between the forward displacement of CoP and stance knee flexion, and between the stance-knee flexion and ML CoP displacement, respectively.

Figure 5A shows a stick diagram of the lower limbs taken at different time-intervals in the sagittal plane. The trial seen in this Figure is the same Medium stance-width trial used in Figure 4. Figures 5B,C show the time-course of the knee AP position of the stance and swing leg, respectively. Figures 5D,E show the time-course of the knee angle of the stance leg and swing leg, respectively. The dots on the curves represent the moment of maximal CoP displacement. As can be seen in the Figure, at t0, i.e., while still standing, both knees are positioned behind the vertical position of CoM, a circumstance that lets gravity extend the knees. Following t0, the stance-leg knee is displaced forward so that it moves anteriorly to the CoM, favoring gravity to flex the knee. Meanwhile, the swing-leg knee is more or less stationary. The stance-leg knee remains anterior to the CoM during APA. Following foot-off of the swing leg, the CoM advancement places the CoM in front of the stance-leg knee so that the action of gravity halts the knee flexion or in some cases extends it slightly.

The grand mean and standard deviation of the AP displacements and flexion angles of the knees at the moment of maximal CoP displacement, relative to the initial position (at t0), are provided in Figures 5F,G. The ANOVA showed a difference in the forward displacement between the knees of the stance leg and the swing leg (*F*_(1,13)_ = 57, *p* < 0.001). Knee forward displacement differed across the three conditions (*F*_(2,26)_ = 66.2, *p* < 0.001). A significant interaction between swing- and stance-leg knee forward displacement and initial stance width was also found (*F*_(2,26)_ = 14.15, *p* < 0.001). The knee angle of the swing leg was significantly different from that of the stance leg (*F*_(1,13)_ = 210.4, *p* < 0.001). There was a general effect of condition on knee angle (*F*_(2,26)_ = 104.2, *p* < 0.001). A significant interaction between swing and stance knee angle and initial stance width was also found (*F*_(2,26)_ = 42.3, *p* < 0.001).

*Post hoc* analysis showed that both forward displacement and the flexion angle of the stance-leg knee were significantly different across conditions (*p* < 0.001). Amplitudes of forward displacement and flexion of the stance-leg were lower for the Small condition and greater for the Large condition compared to Medium (*p* < 0.001). A linear relationship was found between the forward displacement and the flexion angle of the stance-leg knee (*r*^2^ = 0.52, Figure 5H). Finally, a linear relationship was found between the maximum displacement of CoP and the knee flexion of the stance leg (*r*^2^ = 0.64, Figure 5I).

### EMG Activity During Gait Initiation

**Figure 6:**
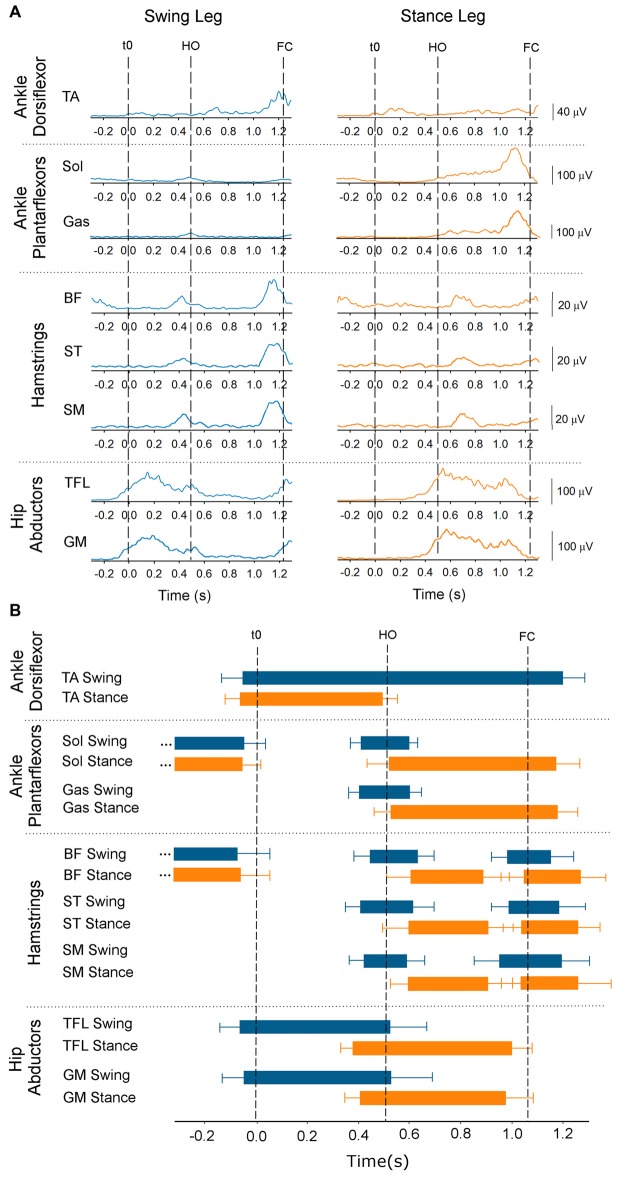
**Onset and offset of EMG activity.** The figure provides temporal information about the ankle dorsi- and plantar-flexors, knee flexors and hip abductor EMG activity. Panel **(A)** shows the average time-course of one subject (Medium condition) of the rectified and low-pass filtered EMG traces (Fcut = 25 Hz) of the tibialis anterior (TA), soleus (Sol), gastrocnemius medialis (Gas), biceps femoris (BF), semitendinosus (ST), semimembranosus (SM), tensor fasciae latae (TFL) and gluteus medius muscles of the swing side (blue, left) and stance side (orange, right). t0, HO and FC are indicated by the dashed lines. Panel **(B)** shows the grand means and standard deviations of all subjects of the onset and offset across the three conditions of the same muscles’ activity calculated with respect to t0.

Figure 6A shows the time-course of the average traces of the TA, soleus, gastrocnemius medialis, BF, ST, semimenbranosus, TFL and gluteus medius from both legs obtained from one subject during the Medium stance-width condition. The grand mean and standard deviations of the onset and offset of the same muscles (across conditions) are shown by the horizontal bars in Figure 6B. When standing, both soleus and BF were active. About 40 ms prior to t0 both soleus and BF were silenced in both legs while TA was activated bilaterally, concurrently with the swing-leg TFL. The offset of the EMG activity in the stance-leg TA and swing-leg TFL roughly coincided with the moment of HO. Triceps surae and hamstring muscles of the stance leg remained silent throughout the APA phase. The swing-leg TA showed two distinct bursts during the step execution phase that lasted until about 100 ms following FC. A brief increase in EMG activity of the triceps surae, hamstrings and TFL of the swing leg was detected about 100 ms prior to HO and ended around the same time as foot-off. During step execution, the beginning of triceps surae activity of the stance leg was recorded slightly prior to HO and ended about 100 ms following FC. The stance-leg TFL was activated about 150 ms prior to HO and was silenced slightly prior to FC. Finally, hamstrings of the stance leg showed a small burst about 100 ms long following HO that usually ended at about mid-single-stance.

**Figure 7:**
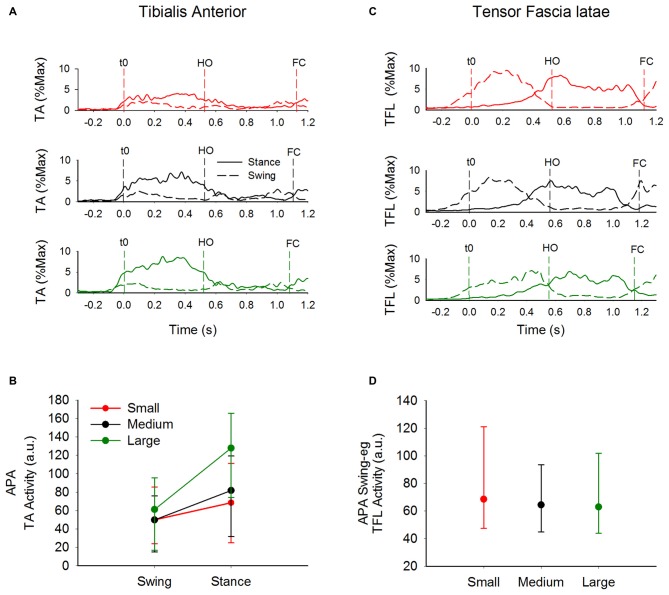
**EMG activity of TA and TFL.** This figure contains information about the activity of tibialis activity and TFL muscles of both sides during GI. Panels **(A,C)** show the average envelope traces of TA and TFL, respectively, of a single subject in the three conditions (from top to bottom: Small, Medium and Large) of the swing leg (dashed line) and the stance leg (solid line). Panels **(B,D)** show the medians along with the 25th and 75th percentiles of the amplitudes of TA activity of the swing and stance legs and of TFL activity of the swing leg during APA, respectively (values are normalized to maximum isometric contraction). TA activity of the swing leg during APA was not affected by the initial stance width. However, TA activity of the stance leg was larger the greater the initial stance-width. The amplitude of TFL EMG activity was only moderately affected by the initial stance width.

Since hamstrings and gastrocnemius muscles are silent during APA, the only candidate responsible for bringing the stance knee forward and initiate knee flexion is the TA muscle that is active during APA. The time-course of the average traces of TA EMG activity of the swing and stance leg of a single subject is provided in the three upper panels of Figure 7A. Figure 7B shows the medians and the 25th and 75th percentiles of the amplitude of the EMG activity of the TA activity (with respect to maximum isometric contraction) of the swing leg and stance leg during APA. The Friedman repeated-measures test showed that the activity of TA was in general significantly lower in the swing- than stance-leg during APA (*χ*^2^ = 24.4, *p* < 0.001). The activity of the TA muscle of the swing leg did not differ across the three conditions (*χ*^2^ = 1.28, *p* = 0.53). However, starting to walk from different initial stance width had an effect on the activity of the stance-leg TA muscle (*χ*^2^ = 28, *p* < 0.001). The Wilcoxon matched pair test indicated that the lowest activity of the stance-leg TA muscle was obtained in the Small condition (*p* < 0.05 for both comparisons) while the greatest activity was achieved in the Large condition (*p* < 0.05 for both comparisons).

Figure 7C shows the time-course of the average traces of the TFL of the swing- and stance-leg during GI in a single subject. The medians and the 25 and 75 percentiles of the amplitude of EMG activity (with respect to maximum isometric contraction) of the swing-leg TFL muscle during APA are shown in Figure 7D. The Friedman repeated-measures test showed no effect of initial stance width on the amplitude of EMG activity of the swing-leg TFL (*χ*^2^ = 4.43, *p* = 0.11) and gluteus medius (*χ*^2^ = 2.6, *p* = 0.09) during APA. Furthermore, ANOVA showed no difference across conditions in the amplitude of EMG activity during the step execution phase of soleus (*F*_(2,26)_ = 0.36, *p* = 0.70), gastrocnemius (*F*_(2,26)_ = 1.02, *p* = 0.37), TFL (*F*_(2,26)_ = 1.97, *p* = 0.16) and gluteus medius (*F*_(2,16)_ = 1.53, *p* = 0.25) of the stance leg.

**Figure 8:**
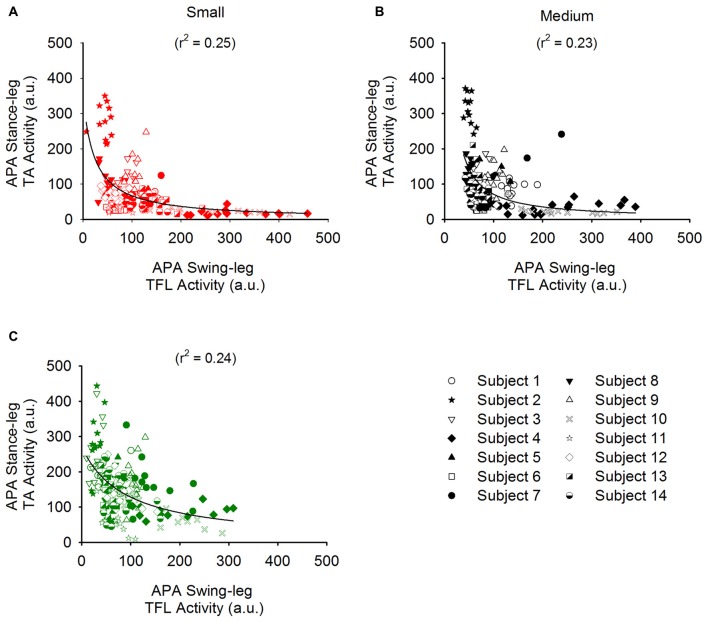
**Relationship between the EMG activity of the TA of the stance leg and that of the TFL of the swing leg.** Panels **(A–C)** show the amplitude of the stance-leg TA activity plotted against that of the swing-leg TFL for all the trials of all the subjects in the Small (red), Medium (black) and Large (Green) stance-width conditions, respectively. Each subject is symbolized by a different icon. A hyperbolic *(y = ab/(b + x))* function was found to bind the activity of these muscles.

**Figure 9:**
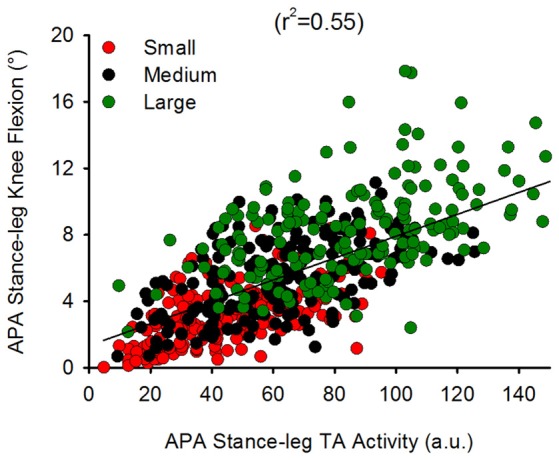
**Relationship between stance-leg TA EMG activity and knee flexion.** This shows a scatter plot of the stance-leg knee flexion angle plotted against the amplitude of stance-leg TA activity during APA (normalized to maximum isometric contraction). A linear relationship was found to bind the two variables (*y* = 0.07*x* + 1.34) suggesting that stance-leg knee flexion might be modulated through TA activity of the same leg.

We also investigated the complementary contribution of hip abduction of the swing side and of knee flexion of the stance side during APA. Figure 8 shows the TA activity of the stance leg plotted against that of the swing-leg TFL (both normalized to maximum activity during isometric contraction) during APA in the three conditions. A hyperbolic relationship interpolated the data points in the Small (*r*^2^ = 0.25), Medium (*r*^2^ = 0.23) and Large condition (*r*^2^ = 0.24). By looking closely into Figure 8, one can also see that the hyperbolic relationship is partly due to the fact that some subjects preferred the hip abductor activity more than stance-knee flexion and vice versa. Figure 9 shows a scatter plot of the amplitude of the stance-leg TA activity from onset until the peak of CoP displacement and the stance-leg knee flexion at the peak of CoP displacement. A linear relationship was obtained between tibialis activity and knee flexion (*r*^2^ = 0.55).

### Disequilibrium Torque During Gait Initiation

**Figure 10:**
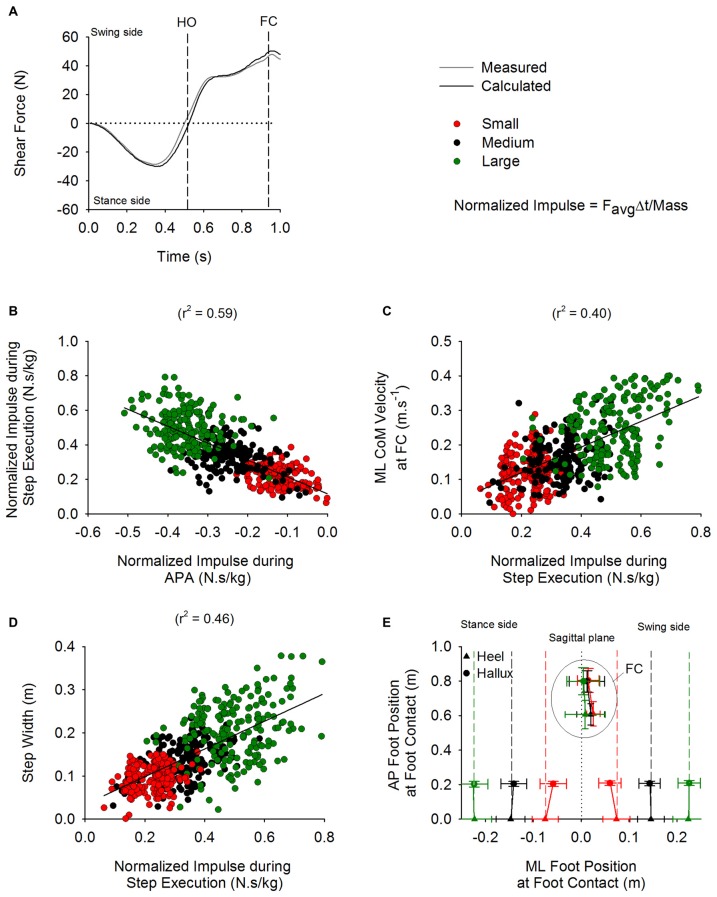
**ML disequilibrium torque and step execution parameters.** The time-course of the shear force calculated from the disequilibrium torque (black trace) and measured from the platform (gray trace) are shown in panel **(A)** (average of 15 trials). The vertical dashed lines indicate HO and FC events. The negative values during APA indicate that the torque is pushing the body in the direction of the stance side. The positive values during step execution designate that the torque is acting in the direction of the swing leg. Panel **(B)** shows a scatter plot representing a linear relationship between the normalized (divided by mass) impulse calculated during APA and the normalized impulse computed during the step execution (*y* = −0.98*x* + 0.12). Panel **(C)** shows a scatter plot representing the linear relationship that was found between the normalized impulse during step execution and the instantaneous ML CoM velocity measured at FC (*y* = 0.37*x* + 0.05). Panel **(D)** shows a scatter plot representing the linear relationship found between the normalized impulse calculated during step execution and step width (*y* = 0.32*x* + 0.03). Panel **(E)** shows the grand means and standard deviations of the position, with respect to the sagittal plane (dotted line), of the heel and hallux markers in ML and anteroposterior (AP) direction at t0 and at FC in the three conditions. As can be seen, the subjects placed the foot at about the same position and with the same orientation close to the sagittal plane at FC in all three conditions.

Figure 10A shows the average time-course of the shear force calculated by dividing the disequilibrium torque by the instantaneous height of the CoM (black line). For comparison, the gray line indicates the average time-course of the shear force measured by the platform. During APA, the shear force produced by the distance between CoP and CoM in the frontal plane displaced the CoM in the direction of the stance leg (negative values in the Figure). Following HO of the swing leg, the torque started acting in the opposite direction.

The impulse theorem allows to quantify the amount of force generated during a certain time interval. Therefore, the impulse derived from the shear force calculated from the disequilibrium torque (see “Materials and Methods: Analysis of Biomechanical Variables” Section) has been used to describe the effect of the torque during APA and step execution on the kinematics of GI in the frontal plane. A linear relationship was obtained between the impulse values calculated from the shear force during APA and those calculated during step execution (*r*^2^ = 0.59; Figure 10B). In addition, the impulse calculated during step execution was correlated with the instantaneous velocity of CoM (*r*^2^ = 0.4; Figure 10C) and step width at FC (*r*^2^ = 0.46; see Figure 10D).

Figure 10E shows the grand mean and standard deviation of the AP and ML position of the heel and hallux markers at the instants of t0 and FC. The AP position of the markers has been calculated relative to the position of the heel markers at t0 (both feet on ground). The ML position of the markers was computed with respect to the subjects’ sagittal plane, calculated as the plane passing between the two anterior superior iliac spines slightly prior to t0. The position of the heel and the hallux markers at FC with respect to the medial line were not different across the three conditions (*F*_(2,26)_ = 2.27, *p* = 0.12 and *F*_(2,26)_ = 0.33, *p* = 0.72, respectively). Step length was also comparable across the three conditions (*F*_(2,26)_ = 0.21, *p* = 0.81).

## Discussion

Human gait is a state of controlled disequilibrium, in which anticipating the position of the body with respect to the ground is critical to avoid destabilizing torques (Bauby and Kuo, 2000). It is fragile, because it can be disturbed by many bodily conditions, including even minor degradations of the sensory information or motor output or both (Mazzaro et al., 2005; Nardone et al., 2014), as well as by cognitive tasks (Lajoie et al., 1993). Further, unlike the hopping gait of kangaroos that is characterized by symmetrical leg jumping action, human bipedal gait is asymmetrical and involves alternate single-support and double-support phases. Hence, the asymmetry of human gait requires substantial balance control in the frontal plane in order to prevent lateral falls (Thorstensson et al., 1984; MacKinnon and Winter, 1993; Orendurff et al., 2004; Sozzi et al., 2013; Wang and Srinivasan, 2014).

During GI, the CNS is confronted with the difficult task of transiting between a symmetrical quasi-static state to an asymmetrical dynamic state (Delafontaine et al., 2015). Indeed, to start walking appropriately, the CoM is moved to a position favorable to gait prior to lifting the future swing leg (Carlsöö, 1966; Brenière and Do, 1986; Brenière et al., 1987; Brenière and Do, 1991; Jian et al., 1993; Elble et al., 1994). On the one hand, the backward shift of CoP, which is responsible for the forward movement of the CoM, is caused mainly by activation of TA and decrease in soleus activity (Crenna and Frigo, 1991; Lepers and Brenière, 1995; Brenière, 2001; Honeine et al., 2013, 2014). On the other hand, the initial lateral shift of CoP in the direction of the swing leg causes the CoM to move toward the stance leg. Carlsöö (1966) and Winter (1995) argued that swing-leg abductor activity during APA unloads the stance leg and loads the other which in turn produces a lateral displacement of the CoP. However, flexing the knee of the stance leg during APA can also help unloading the swing leg. It has been already shown that the knee is moderately flexed in the stance leg during walking. This knee flexion prevents higher rate of loading during gait, and translates to attenuated rates of loading and peak vertical ground reaction force in healthy adults (Riskowski et al., 2005; Riskowski, 2010). Hence, we hypothesized that the stance-leg knee flexion, which is part of the normal motor program for walking, could also be exploited in order to unload the stance leg during the preparation of GI.

### Knee Flexion of the Stance Limb During APA

In line with Lyon and Day (1997), initiating gait at increasing stance widths causes greater displacement of the CoP in the direction of the swing leg and CoM in the opposite direction during APA. Our results also confirm the findings of Carlsöö (1966) and Winter (1995) in that the CoP displacement during APA is caused by unloading and loading the stance and swing legs, respectively (see Figure 4). In our hands, the greater excursions of CoP and CoM occurred without significant modifications of the global kinematic variables of GI (see Table 1) and most notably the duration of APA. This facilitates the comparisons across conditions without the need to normalize in time.

As can be seen in Figure 5, at t0 both knees are placed behind the vertical projection of CoM. However, during APA, the stance knee is displaced forward, placing the joint anterior to the CoM vertical projection in the sagittal plane allowing gravity to participate in flexing the knee. Meanwhile, the swing-leg knee position and angle remain more or less constant. The stance-leg knee forward advancement and flexion augmented with increasing stance width. The amplitude of the flexion of the stance-leg knee was also correlated with the lateral displacement of CoP. The linear relationship between stance-leg knee flexion and lateral displacement of CoP is in line with our hypothesis that stance knee flexion unloads the stance leg and thus participates in shifting the CoP in the direction of the swing leg.

Hamstrings and gastrocnemius activity are associated to knee flexion during the double stance phase of gait (Goldberg et al., 2004). However, none of those muscles were active during flexion of the stance-leg knee in early APA. Moreover, BF that shows tonic activity while standing becomes silent during APA (see Figure 6). The reason might be that hamstrings also extend the hip. Hip extension during GI should potentially slightly restrict forward movement and is therefore undesirable. Conversely, the ankle plantarflexion produced by gastrocnemius activation would impede the backward CoP displacement required for generating forward motion. Slightly prior to t0, soleus and BF muscles are silenced bilaterally followed by TA activation (Figure 6). As more appropriately stated by Zajac and Gordon (1989), “at times, such as in standing, muscles crossing the ankle extend (soleus) or flex (TA) the knee much more that they flex and extend the ankle”. In this study, the amplitude of the TA muscle of the stance leg is greater than that of the swing leg. Furthermore, the amplitude of the stance-leg TA activity increases with larger stance-widths (see Figure 7). This result suggests that the reduction in soleus tonic activity and the activation of the TA of the stance leg move the stance-leg knee forward with respect to the CoM position in the sagittal plane, thereby favoring gravity to flex the stance knee. Indeed, a linear relation between the amplitude of EMG activity of TA of the stance leg and flexion of the stance-leg knee is found here (Figure 9), strengthening the hypothesis that the stance-leg TA participates in displacing the CoP in the direction of the swing leg during the preparation phase of GI. It is to be noted that feet plantar afferents can influence the activity of TA and soleus during APA when raising one leg quickly (Do and Gilles, 1992). Cutaneous afferents can sense the augmentation in the shear force under the feet (Abbruzzese et al., 1996; Choi et al., 2016) that occurs with increasing stance width (Carmines and MacMahon, 1992). The changes of the cutaneous afferent input, in the different conditions, could have contributed to the modulation of stance-leg TA activity that participates in the lateral displacement of CoP during APA.

Following lift-off of the swing foot, the forward momentum of CoM places the latter anterior to the stance-leg knee, as occurs during the single-support phase of steady-state gait (Liu et al., 2008). This causes gravity to extend the stance knee thereby halting the flexion that occurred during APA. Furthermore, the activity of triceps surae and the hamstring muscles, increases during step execution. Those muscles could also be implicated in checking the knee flexion during single support.

### The Complementary Action of Hip Abduction and Stance Knee Flexion

Starting gait with different stance widths has no significant effect on the amplitude of hip abductor EMG activity of the swing leg during APA, albeit a slight tendency of some subjects (9 out of 14) to decrease hip abductor activity was observed when starting gait from a large stance width. Remarkably, a hyperbolic relationship binds the amplitude of stance-leg TA activity with that of the hip abductor muscles of the stance leg in each of the three conditions (see Figure 8). Some subjects relied more on swing-leg hip abductor activity and others on stance-leg dorsiflexor activity, while most relied on both rather equally. It is to be noted that the decrease in hip abductor activity in the Large condition of the nine subjects mentioned above is accompanied by a systematic increase in stance-leg knee flexion. A possible explanation is that due to biomechanical constraints, the hip abduction strategy is less efficient when starting from a large stance width. Conclusively, the actions of stance-knee flexion and swing-leg hip abduction appear to be complementary and synergistic in unloading the stance leg and loading the swing leg during APA. The coordination of the two strategies would result in more efficient and flexible ML displacement of the CoP in the direction of the swing leg.

### The Effects of Modulation of ML CoP Displacement During APA on the Execution of the First Step

By modulating hip abduction and stance-leg knee flexion during APA, the CNS efficiently controls the distance between CoP and CoM in the frontal plane and consequently the disequilibrium torque during APA. Moreover, due to friction between the floor and the stance foot, the torque is converted into shear force that in turn displaces the CoM and places it just medially with respect to the stance foot during the single-stance phase of the execution of the first step (see Figure 3). In line with Lyon and Day (2005), our results show that the subjects modulate the CoP displacement during APA across conditions in order to control the ML distance between CoM and CoP during the step-execution phase. More precisely, the small displacements of CoP during the Small stance-width condition produce a small ML CoM-CoP distance during step execution, while the ample displacements of CoP during the Large stance-width condition produce a greater ML CoM-CoP distance (see Figure 3). Consequently, the ML disequilibrium torque and the CoM velocity during step execution are greater when larger the initial stance-width. The greater velocities of the CoM during step execution measured under larger initial stance-width causes the stepping foot to land at a larger distance with respect to the stance foot (Caderby et al., 2014). This causes the foot to be placed close to the subjects’ mid-sagittal plane at the FC in all three conditions, so that subjects subsequently progressed easily along their initial straight-ahead line (see Figure 10). This result is interesting, because it shows that, already at the first step, subjects tend to place the foot in the appropriate position for performing the next sets, regardless of the initial postural attitude. In Honeine et al. (2014) we showed that by modulating the CoM and CoP distance in the sagittal plane the CNS sets step length, the antero-posterior velocity of CoM and the duration of the first step. Here, we complete those results by showing that by controlling the disequilibrium torque in the frontal plane during APA, the CNS determines also the ML velocity of CoM and the ML position of the swing leg at the instant of first FC.

### Limitations

The initial stance width for each condition (15, 30 and 45 cm) was fixed for all the subjects. Due to anthropometric differences between subjects, a same width of the base of support could provoke different biomechanical constraints on the CoP displacement. Nonetheless, possibly because of the large inter-subject variability, the maximum displacement of the CoP towards the swing leg during APA was not significantly correlated with height or width of the pelvis, measured as the distance between the two anterior superior iliac spines.

Furthermore, the results of this study do not permit to make a quantitative statement about which strategy (hip abduction or stance-leg knee flexion) is mechanically more effective in displacing the CoP towards the swing leg during APA. To do so, in our opinion, this would require the calculation the contribution of the hip abduction and knee flexion torques to the CoP displacement, which is beyond the scope of this current article. One would also consider that different initial stance widths can slightly modify the angle of the tendon insertion between the hip abductors and femur. Therefore, different abduction forces could be generated for the same level of EMG activity, so that comparing EMGs across condition should be done with care.

### Final Considerations

Our results support the hypothesis that both hip abduction and stance-leg knee flexion participate in unloading the stance leg and displacing the CoP in the direction of the swing leg during GI. On the one hand, hip abduction is caused by activation of the hip abductors of the swing leg (Carlsöö, 1966; Winter, 1995). On the other hand, stance-leg knee flexion is favored by the activation of TA and silencing of soleus of the same leg. The flexion of the knee during APA could be seen as an adaption of the CNS to facilitate and increase the robustness of the ML balance control process. Consequently, when ML balance control is examined in patients suffering from motor problems during GI, both hip abduction and stance knee flexion should be taken into account, in addition to initial stance width (Goodworth et al., 2014). Knee-flexion control in the frontal plane during APA could be inadequate in patients suffering from gait problems such as cerebral palsy, Parkinson’s disease (Hiraoka et al., 2006; Hiraoka and Abe, 2007; Okada et al., 2011a,b; Mazzone et al., 2014), stroke, amputees (Aruin, 2016). For instance, freezing of gait in Parkinsonian patients is associated with knee trembling (Yanagisawa et al., 1991; Ueno et al., 1993; Hausdorff et al., 2003; Schaafsma et al., 2003; Bloem et al., 2004; Moore et al., 2008). Jacobs et al. (2009) found that during GI, knee trembling causes multiple APAs that are observable as a right-left leg loading-unloading cycles. Interestingly, the alternating unloading and loading of the legs was accompanied by similar alternating activation and deactivation of right-left TA (see Figure 2 in Jacobs et al., 2009). Therefore, knee trembling in Parkinson’s disease patients might be preventing them from displacing CoM correctly towards the stance leg and thus not allowing them to initiate gait properly. Indeed, the smaller ML CoP displacement during APA and larger step width of the first step at GI have been observed in Parkinson’s disease (Okada et al., 2011a,b). This could be in part associated with inappropriate knee flexion. Therefore, correcting the knee flexion angle of the stance leg during APA with a smart orthosis could possibly be an effective solution for enhancing GI and possibly steady-state gait in these patients.

## Author Contributions

J-LH contributed with project creation, data collection, data analysis and drafted the manuscript. MS contributed with project creation, data analysis. OC contributed in data collection, data analysis. M-CD contributed with project creation and data analysis. All authors discussed the results and participated in the revision of the manuscript.

## Funding

This study was supported in part by the Ricerca Finalizzata grants (GR-2009-1471033 and RF-2011-02352379) from the Italian Ministry of Health and by the PRIN grants (2009JMMYFZ and 2010MEFNF7) from the Italian Ministry of University.

## Conflict of Interest Statement

The authors declare that the research was conducted in the absence of any commercial or financial relationships that could be construed as a potential conflict of interest.
